# 

**Published:** 1900-01

**Authors:** 


### CONTENTS:

### SELECTIONS.

Article I.—Interstate Comity in Dental Legislation. Rodrigues Ottolengui, M. D. S. .... 385

" II.—Bleaching Devitalized Teeth With Pyrozone. J. P. Parker, D. D. S .... 410

" III.—A Case of Carcinoma of the Lower Jaw—Operation. Reported by A. Frank Kerns .... 416

" IV.—Reminiscences of Dr. Bonwill. Eugene S. Talbot, M. D., D. D. S .... 419

" V.—Dental Fees .... 422

" VI.—Dental Science in 1480—As Expounded by Peter de Largelata. William H. Trueman .... 424

" VII.—Poor Man's Crown. Dr. J. N. Davenport .... 426

### MONTHLY SUMMARY.

How to Remove a Pin Cemented to a Root .... 428

Compressed Cork and Its Uses .... 428

Points in Favor of the Use of Alcohol and Their Refutation .... 429

An Evil and Its Remedy .... 430

Paralysis Following General Anæsthesia .... 431

New Method of Treatment of Wounds .... 431

